# Identification of a Tertiary Lymphoid Structure Signature for Predicting Tumor Outcomes Through Transcriptomics Analysis

**DOI:** 10.3390/genes17020239

**Published:** 2026-02-16

**Authors:** Mengdi Zhou, Fangliangzi Meng, Fan Wu, Chi Zhou

**Affiliations:** 1 Shanghai Immune Therapy Institute, Renji Hospital, Shanghai Jiao Tong University School of Medicine, Shanghai 200127, China; mdzhou@innostarbio.com; 2 Shanghai InnoStar Bio-Tech Co., Ltd., Shanghai 200092, China; 3 Bioinformatics Department, School of Life Sciences and Technology, Tongji University, Shanghai 200092, China; 1911007@tongji.edu.cn

**Keywords:** cancer genomics, tertiary lymphoid structures, meta-analysis, tumor prognosis, single-cell sequencing, spatial transcriptome

## Abstract

Background: Tertiary lymphoid structures (TLSs) play a crucial role in regulating tumor invasion and metastasis and serve as a promising prognostic biomarker in immunotherapy, influencing survival and immune response in multiple cancers. However, existing studies rely on limited gene signatures to assess TLSs, and there remains a lack of comprehensive TLS-related features for pan-cancer prognosis or immunotherapy response prediction. Methods: Based on published TLS gene signatures, mutation data, and expression profiles from 33 tumor types in TCGA, along with data from 15 immune checkpoint blockade (ICB) cohorts, we first systematically evaluated six TLS gene signatures in relation to immune-related indicators and assessed their predictive and prognostic performance across tumors and immunotherapy. Subsequently, using meta-analysis, we constructed a de novo TLS-related gene feature set, termed predictTLS, designed to predict ICB efficacy and prognosis. The rationality and effectiveness of predictTLS were validated using internal validation sets, single-cell transcriptomic, and spatial transcriptomic data. Results: The evaluation revealed associations between TLS gene signatures and key immune-related indicators. The newly constructed predictTLS feature set demonstrated effectiveness in predicting both ICB therapy outcomes and patient prognosis across the analyzed cohorts. Validation across internal datasets, single-cell profiles, and spatial transcriptomics supported the robustness and biological relevance of predictTLS. Conclusions: This study provides a systematically validated, de novo TLS-related gene signature that can serve as a clinical biomarker for predicting immunotherapy response and prognosis in pan-cancer settings. These findings offer new tools for risk stratification and potential therapeutic targeting in tumor immunotherapy.

## 1. Introduction

As cancer progresses, tumor cells experience genomic, epigenetic, and morphological alterations within particular tissues before they start metastasizing to other organs [1,2,3]. Accumulated genetic mutations in tumor cells result in the expression of tumor antigens, which activate both innate and adaptive immune responses to target and eliminate the cancer cells [4,5]. Typically, effective adaptive immune responses against cancer occur in secondary lymphoid organs (SLO) [6]. However, further research on the tumor microenvironment (TME) has revealed the mechanisms underlying the generation and regulation of anti-tumor immune responses. Studies have shown that anti-tumor immune responses occur not only in SLO but also directly within organized cellular aggregates at the tumor site. These aggregates structurally resemble SLO and are analogous to secondary lymphoid structures. This cluster has been named tertiary lymphoid structures (TLSs). TLSs, as a site for the generation of circulating effector immune cells, play a crucial role in inhibiting tumor growth within the TME [7,8,9].

Recent evidence indicates that tertiary lymphoid structures (TLSs) are crucial in regulating tumor invasion and metastasis. A beneficial effect of TLS density on overall survival and disease-free survival has been observed in patients, regardless of the detection method used on tumor tissue sections, of lung cancer, colorectal cancer, pancreatic cancer, oral squamous cell carcinoma, and invasive breast cancer [10,11,12,13,14,15,16,17]. This prognostic impact was independent of pathological tumor-node-metastasis (TNM) staging for lung, colorectal and pancreatic cancers. Despite a correlation between densities of TLSs and infiltrating T cells, the favorable prognostic value of both parameters was found to be independent in colorectal cancer [12]. Recent studies have also demonstrated that the enrichment score of tertiary lymphoid structure (TLSs) characteristics can serve as a promising prognostic biomarker in immunotherapy. The presence of abundant tertiary lymphoid structures is an independent prognostic factor for HR^−^/HER2^+^ breast cancer patients who have received adjuvant chemotherapy and trastuzumab treatment [18]. Furthermore, a high score for TLS’s characteristics is significantly correlated with improved overall survival and immune response in patients with melanoma and non-small cell lung cancer who have undergone PD-1 or CTLA-4 immune checkpoint inhibitor therapy [19,20]. Patil et al. utilize scRNA-seq of NSCLC tumors to identify three main populations of intratumoral B and plasma cells. Deconvolution of bulk RNA-seq of two large, randomized NSCLC clinical trials demonstrates a strong association of increased intratumoral plasma cells with longer overall survival in patients treated with PD-L1 blockade, but not with chemotherapy [20]. Interestingly, through spatial transcriptomics data, Meylan et al. show that tertiary lymphoid structures found in tumors are sites of generation of fully mature B cell immunity. Plasma cells disseminate into tumor beds, producing antibodies that bind to tumor cells and initiate their apoptosis, providing a mechanism to support cancer immunotherapies that modulate the tumor microenvironment [21].

However, in the aforementioned studies, the gene signatures used to assess tertiary lymphoid structures (TLSs) were limited, such as the use of only 12 chemokines, which cannot effectively characterize TLSs’ features and functions [15]. Additionally, researchers have only evaluated their association with tumor prognosis and immune therapy in limited tumor types. Currently, there is still a lack of effective and comprehensive TLS features for prognosis or response prediction in immunotherapy at a pan-cancer level. Here, based on published TLSs’ feature gene sets, mutation information, and expression profiles from 33 tumor types in The Cancer Genome Atlas (TCGA), as well as data from 15 immune checkpoint blockade (ICB) therapy cohorts, we first systematically evaluated the association of six TLS gene signatures with immune-related indicators, as well as their predictive and prognostic performance in immune therapy. Subsequently, using meta-analysis methods, we constructed a de novo TLS-related gene feature, i.e., predictTLS, that can effectively predict the efficacy of ICB therapy and prognosis. We validated the rationality and effectiveness of this de novo gene signature using independent validation cohorts of patients treated with immune checkpoint blockade, single-cell transcriptome data, and spatial transcriptome data. These results provide new clinical biomarkers and potential therapeutic targets for tumor immunotherapy.

## 2. Materials and Methods

### 2.1. Systematic Review of Published TLSs’ Prognostic Signatures

We initiated an electronic search on PubMed using the terms “TLSs signature”, “tumor or cancer”, and “immunotherapy”. Studies published in English before October 2022 were manually screened for eligibility. Publications featuring fewer than three signature genes were excluded, resulting in the identification of six TLS-associated signatures (Supplementary Table S1).

### 2.2. Data Acquisition and Processing

Mutation profiles, gene expression data, and survival information for 33 TCGA cancer types were obtained from the UCSC Xena database (http://xena.ucsc.edu/ (accessed on 9 May 2025)). Gene expression data consisted of log2(x + 1) transformed RSEM-normalized counts. Gene identifiers were aligned to genomic coordinates using the UCSC Xena HUGO probeMap. Curated survival datasets included both overall survival (OS) and progression-free survival (PFS). Tumor mutation burden (TMB), expression profiles, and clinical data from immune checkpoint blockade (ICB)-treated cohorts were collected from previous publications, yielding 15 ICB datasets (Supplementary Table S2A) [22,23,24,25,26,27,28,29,30,31,32,33,34,35,36]. Single-cell RNA sequencing (scRNA-seq) data from eight ICB-treated datasets were sourced from the Tumor Immune Single-Cell Hub 2 (TISCH2) [37] (Supplementary Table S2B). Downstream analysis is performed in R using the Seurat pipeline, which begins with the creation of a Seurat object from the count matrix. Quality filtering is applied to remove low-quality cells based on three key metrics: the number of unique molecular identifiers (UMI > 500), the number of detected genes (250 < nFeature_RNA < 7500), and the percentage of mitochondrial reads (percent.mt < 15%). Doublets are typically identified and removed using additional tools such as DoubletFinder (v2.0.0) prior to integration. After filtering, data normalization is performed using NormalizeData with a log-transformation scale factor of 10,000. Highly variable features (typically 2000 genes) are identified via FindVariableFeatures using the “vst” selection method. The data are then scaled and centered using ScaleData, regressing out unwanted sources of variation such as percent.mt, nCount_RNA, and cell cycle scores if required. Principal component analysis (PCA) is conducted on the scaled data using RunPCA, and the first 30 significant principal components are selected based on elbow plots or jackStraw analysis. For non-linear dimensionality reduction, UMAP and t-SNE are computed using RunUMAP and RunTSNE, respectively, with the same set of principal components. The celltypes annotation was retrieved from the original studies. Treatment response was classified according to RECIST v1.1: responders (R) included patients with complete response, partial response, or stable disease without PFS events within 6 months; non-responders (NR) were those with progressive disease or stable disease accompanied by a PFS event within 6 months. If RECIST criteria were unavailable, PFS status at 6 months was used to classify R and NR. Patients with stable disease but missing PFS data were deemed non-assessable. scRNA-seq data were processed and visualized using Seurat [38] (v4.0.1). Quality control is performed in R using the Seurat pipeline to filter out low-quality spots based on the number of unique molecular identifiers (nCount_Spatial), number of detected genes (nFeature_Spatial), and percentage of mitochondrial reads (percent.mt), with spot-level thresholds adjusted according to tissue morphology and sequencing depth. Data normalization is conducted using NormalizeData followed by ScaleData, and signature scores for response groups were computed as the GSVA score of 17 PredictTLS genes.

Spatial transcriptomic data from 10X Visium profiles of 24 clear cell renal cell carcinoma (ccRCC) samples (12 frozen, 12 FFPE) were obtained from Meylan et al. [21]. TLS’s presence and localization were annotated by a pathologist following H&E staining. Visium data were processed similarly using Seurat with the assay = “spatial” parameter. Spot-level signature scores were derived as the GSVA score of the 17 PredictTLS genes.

### 2.3. TMB Calculation in TCGA

TMB was defined as the number of non-synonymous mutations (missense, frameshift indels, and stop codons) per megabase (Mb) of sequenced territory (38 Mb for TCGA). For each cancer type, samples were stratified into high or low TMB groups based on the median TMB value.

### 2.4. “Hot” and “Cold” Cancer Type Definitions

Based on the established understanding of tumor immune microenvironments, we have stratified the cancer types in our analysis into “hot” and “cold” categories. This classification primarily reflects the fundamental degree and characteristics of immune cell infiltration within the tumor. Hot Tumors: These cancer types are generally characterized by a higher level of immune cell infiltration, particularly cytotoxic T cells, often indicating a more active but potentially suppressed immune response. This group comprises 18 cancer types, including common malignancies such as Bladder Urothelial Carcinoma (BLCA), Breast Invasive Carcinoma (BRCA), Cervical cancer (CESC), Colorectal Adenocarcinomas (COAD, READ), Head and Neck Squamous Cell Carcinoma (HNSC), Kidney Renal Clear Cell Carcinoma (KIRC), Liver Hepatocellular Carcinoma (LIHC), Lung Adenocarcinoma (LUAD), Lung Squamous Cell Carcinoma (LUSC), Lung cancer (LUNG), Mesothelioma (MESO), Ovarian cancer (OV), Pancreatic Adenocarcinoma (PAAD), Prostate Adenocarcinoma (PRAD), Skin Cutaneous Melanoma (SKCM), Stomach Adenocarcinoma (STAD), and Uterine Corpus Endometrial Carcinoma (UCEC). Cold Tumors: These types typically exhibit an “immune-desert” or “immune-excluded” phenotype, with minimal T cell infiltration into the tumor core. This category encompasses 15 generally rarer or more treatment-resistant cancers, including Adrenocortical Carcinoma (ACC), Cholangiocarcinoma (CHOL), Diffuse Large B Cell Lymphoma (DLBC), Esophageal Carcinoma (ESCA), Kidney Chromophobe (KICH), Kidney Renal Papillary Cell Carcinoma (KIRP), Testicular Germ Cell Tumors (TGCT), Thyroid Carcinoma (THCA), Thymoma (THYM), Uterine Carcinosarcoma (UCS), Uveal Melanoma (UVM), Glioblastoma (GBM), Lower Grade Glioma (LGG), Pheochromocytoma/Paraganglioma (PCPG), and Sarcoma (SARC). This explicit stratification allows us to specifically evaluate the performance of immune-related signatures across these two fundamentally different immune contexts.

### 2.5. Meta-Analysis of Gene Signatures in TCGA and ICB Cohorts

In TCGA cohorts, continuous survival time was analyzed using Cox proportional hazards models (coxph from the R survival package) for each signature in each tumor type. Meta-Analysis hazard ratios (HRs) were calculated by DerSimonian–Laird random-effects meta-analysis models, implemented by the R metagen package, with the parameter random = ‘True’ [39]. The pooled effect estimate $\hat{\theta }$ was calculated as:

$$\hat{\theta }=\frac{\sum_{k=1}^{K}\hat{\theta_{k}}\omega_{k}}{\sum_{k=1}^{K}\omega_{k}};\;\omega_{k}=\frac{1}{s_{k}^{2}}$$

where $\hat{\theta }$ is the HR estimate from study k and $s_{k}$ is its standard error. For ICB cohorts, treatment response was modeled using logistic regression, and regression coefficients were similarly meta-analyzed.

### 2.6. De Novo TLS Signature Construction

Seventy-four genes from ten studies with transcriptomic data were included in a meta-analysis assessing association with immune response (gene list and study references are provided in Supplementary Table S1). Genes with >50% zero expression were excluded. After false discovery rate (FDR) correction, no genes remained significant. Following Shi et al. [40], genes were ranked by log-odds ratio and iteratively included using cutoffs from 0 to 0.5 (step size 0.005). A cutoff of 0.145 yielded the 17-gene PredictTLS signature. The PredictTLS score was defined as the Gene Set Variation Analysis (GSVA) enrichment score of these genes, applied to z-score-transformed expression values.

### 2.7. Immune Signature Scoring

The COX-IS score was computed as previously described [41]. Pro-tumor (CP) and anti-tumor (CI) inflammatory genes were defined as:

$$pos=\sum_{i=1}^{n_{p}}G_{i}^{pos}\left(e\right)$$

$$neg=\sum_{i=1}^{n_{n}}G_{i}^{neg}(e)$$

where CP genes included VEGFA, CCL2, IL8, CXCL1, CXCL2, CSF3, IL6, IL1B, IL1A; CI genes included CCL5, CXCL9, CXCL10, CXCL11, IL12A, IL12B, IFNG, CD8A, CD8B, GZMA, GZMB, EOMES, PRF1, STAT1, and TBX21. COX-IS was calculated as:

$$COX-IS=\frac{\frac{1}{n_{p}}pos}{\frac{1}{n_{n}}neg}$$

Immunophenotype Score (IPS) [42] and Innate Anti-PD-1 Resistance (IPRES) scores [23] were computed as originally described. Z-score normalization was applied before GSVA (v1.42.0) or weighted averaging. Immune infiltration was estimated using CIBERSORT (v1.0.0) [43] with the LM22 reference.

### 2.8. Gene Signature Network and Enrichment Analysis

Protein–protein interactions among PredictTLS genes were analyzed using the STRING [44] database. Functional enrichment analysis was performed with clusterProfiler (v4.2.0) [45].

### 2.9. Correlation Analysis and Statistical Methods

Spearman correlations between signatures were computed within each ICB dataset and summarized as median values across studies, visualized using the corrplot R package (v0.84). Categorical variables are reported as percentages, continuous variables as medians and IQRs. Group comparisons used Fisher’s exact test (categorical) or Wilcoxon rank-sum test (continuous), with permutation testing for small samples (<10). Biomarker associations with immune response were evaluated via logistic regression; survival associations were evaluated using Cox models. Kaplan–Meier curves and log-rank tests assessed PredictTLS stratification. All analyses were performed in R (v3.6.3). *p*-values were adjusted using the Benjamini–Hochberg method (FDR); significance was defined as FDR ≤ 0.05.

## 3. Results

### 3.1. Meta-Analysis Reveals Differences in Prognostic Capabilities of 12 Features Across 33 Tumor Types in the TCGA Dataset

**Figure 1:**
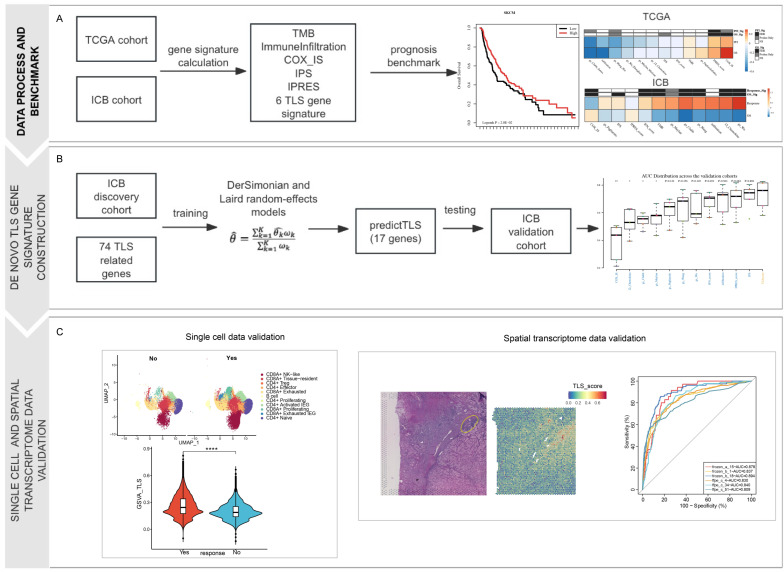
Flow chart for study design. (**A**) Data collection, gene signature collection, and benchmark analysis of 12 signatures based on TCGA data and ICB cohorts. (**B**) De novo TLS gene signature construction by utilizing meta-analysis and efficiency validation on validation cohorts. (**C**) Efficiency validation on single-cell sequencing data and spatial transcriptome data. **** means *p* value < 0.001. ** means *p* value < 0.01. * means *p* value < 0.05.

**Figure 2:**
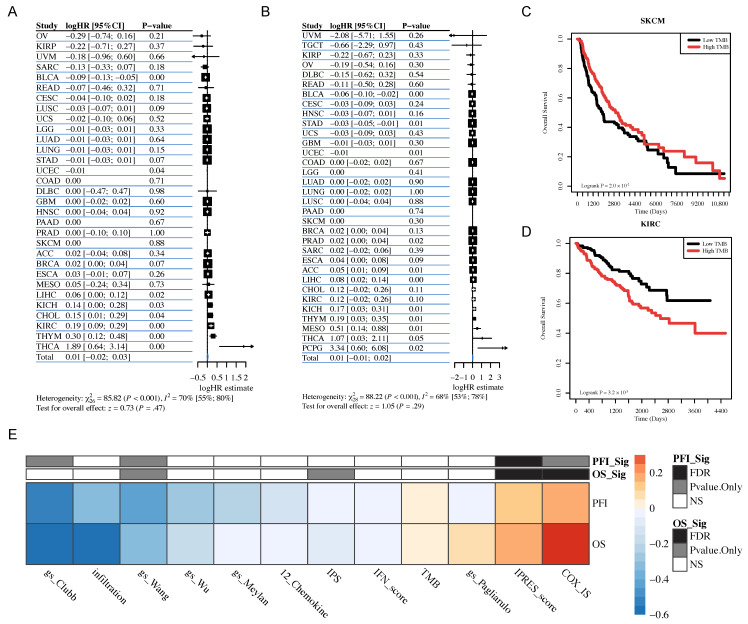

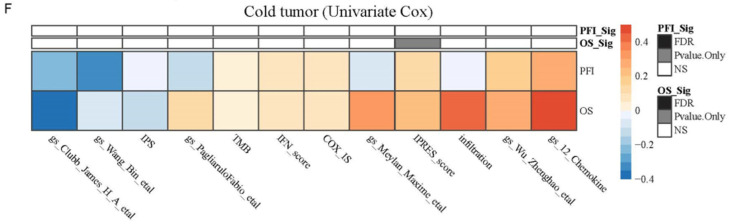
Meta-analysis of all features on TCGA datasets. (**A**) Cox proportional-hazards model and meta-analysis of TMB utilize overall survival (OS) as the outcome variable. (**B**) Cox proportional-hazards model and meta-analysis of TMB utilize progression-free survival (PFS) as the outcome variable. (**C**) Kaplan–Meier plots of overall survival difference between tumors with TMB high and low groups in SKCM. (**D**) Kaplan–Meier plots of overall survival difference between tumors with TMB high and low groups in KIRC. (**E**) Meta-analysis results of 12 features across “Hot” cancer types based on HR derived from the Cox proportional-hazards model. (**F**) Meta-analysis results of 12 features across “Cold” cancer types based on HR derived from the Cox proportional-hazards model.

A total of 12 gene signatures, including 6 TLS-related gene signatures, 5 immune infiltration or cytotoxicity signatures, and tumor mutation burden (TMB), were collected from previous studies (see Material Methods and Supplementary Table S1). We first checked the prognostic capabilities of 12 features across 33 tumor types by utilizing the TCGA dataset (Figure 1, top panel). The results from the univariate Cox regression model indicate that, in BLCA and UECE, high TMB is significantly associated with better OS (Overall Survival) and PFI (Progression-Free Interval) (Figure 2A,B). However, in several tumor types, including LIHC, KICH, THYM, and THCA, high TMB is significantly associated with poorer OS and PFI (Figure 2A,B,D). It is worth noting that, although significance was not observed in the univariate Cox regression model for SKCM, Kaplan–Meier curves suggest that patients with high TMB have longer overall survival, consistent with previous findings from multiple studies (Figure 2C). Many studies have found that tumor patients with high tumor mutation burden (TMB) tend to have better survival outcomes and are more likely to benefit from immune therapies such as immune checkpoint inhibitors [46,47,48]. This may be because a high TMB results in the presentation of more mutated antigens on the surface of tumor cells, making it easier for the immune system to recognize and attack these cells. Subsequently, a meta-analysis of HRs (Hazard Ratios) from Cox models for individual cancer types indicates, according to the fixed-effect model, that TMB does not possess the ability to serve as a biomarker across various cancer types (Test for overall effect: z = 1.05, *p* = 0.29 for PFS and z = 0.73, *p* = 0.47 for OS) (Figure 2A,B). Similarly, we conducted univariate Cox regression analyses for the other 11 features, and the results indicate substantial variations in the association of each feature with survival outcomes across different tumor datasets. We next performed meta-analysis based on the HR derived from Cox models in “hot” cancer types and “cold” cancer types, respectively. The meta-analysis results suggested that well-established immune features such as COX-IS and IPRES can effectively predict patient survival outcomes at the pan-cancer level in “hot” tumors (cancer type level FDR < 0.05 for both OS and PFI) (Figure 2E). In contrast, these published features cannot reliably predict patient survival outcomes at the pan-cancer level in “cold” tumors (cancer type level FDR > 0.05 for all) (Figure 2F). This conclusion remained robust in multivariate Cox analyses that accounted for influencing factors such as gender, age, and tumor stage (Supplementary Figure S1A). Specifically, in multivariate Cox analyses, TMB was significantly associated with better overall survival in BRCA and KICH; IPS was significantly associated with better overall survival in KIRC and KIRP; IPRES_score was significantly associated with better overall survival in STAD and PRAD, and most of the TLS-related signatures were significantly associated with better overall survival in STAD, PRAD, and OV (Supplementary Table S3). These results suggest that due to differences in tumor immune infiltration, a single biomarker is unlikely to effectively predict prognosis at the pan-cancer level. The performance of these biomarkers is markedly different in hot versus cold tumors, indicating that biomarker analysis should be conducted according to the tumor’s immune infiltration subtype. We also noticed the limitations in the current set of TLS-related features, which may not adequately characterize the expression patterns of tertiary lymphoid structures. Therefore, it is necessary to identify new gene features that comprehensively represent tertiary lymphoid structure expression patterns.

### 3.2. Pan-Cancer Testing of 12 Gene Signatures Association with ICB Responses

**Figure 3:**
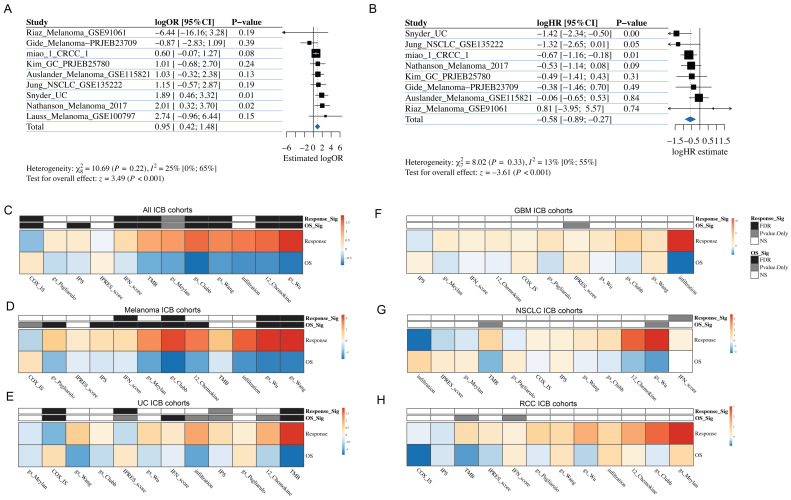
Meta-analysis of all features on ICB cohort datasets. (**A**) Logistic regression model and meta-analysis of TMB on ICB cohort datasets utilize response as the outcome variable. (**B**) Cox proportional-hazards model and meta-analysis of TMB on ICB cohort datasets utilize overall survival (OS) as the outcome variable. (**C**) Meta-analysis results of 12 features across 15 ICB cohort datasets. (**D**) Meta-analysis results of 12 features across melanoma ICB cohort datasets. (**E**) Meta-analysis results of 12 features across urothelial carcinoma ICB cohort datasets. (**F**) Meta-analysis results of 12 features across GBM ICB cohort datasets. (**G**) Meta-analysis results of 12 features across NSCLC ICB cohort datasets. (**H**) Meta-analysis results of 12 features across RCC ICB cohort datasets. All the analyses were performed based on the hazards ratio (HR) derived from the Cox proportional-hazards model and the odds ratio (OR) derived from the logistic regression model.

Next, we evaluated the association of 12 gene signatures with survival outcomes or response to ICB treatment in ICB cohorts (Figure 1, top panel). All the curated signatures were z-scaled and transformed to allow for effect-size comparison. Of note, contrary to the TCGA dataset, the meta-analysis of Cox models revealed that TMB was significantly associated with clinical outcomes at the pan-cancer level after correction for multiple testing (both FDR < 0.05 for response and OS) (Figure 3A,B), while well-established immune features were not (at least one FDR > 0.05). Interestingly, three out of six TLS-related signatures were more significantly associated with clinical outcomes than tumor mutation burden, with larger OR for response or larger HR for OS, and smaller FDR (Figure 3C, Supplementary Figure S1B). Subgroup analysis grouped by cancer types showed substantial heterogeneity of predictive association between gene signatures and clinical outcomes. Our results revealed similar patterns of predictive association of gene signatures in melanoma and urothelial carcinoma (UC) (Figure 3D,E). In NSCLC tumors, however, several signatures such as infiltration, IPRES, and COX_IS displayed an opposite predictive association compared to pan-cancer or melanoma. For instance, COX_IS and gs_Meylan were associated with better outcomes while TMB was associated with worse overall survival in the meta-analysis, although not significant (Figure 3G). Immune cell infiltration was demonstrated to be a strong predictor of clinical outcomes in GBM, which was not observed in other cancer types (Figure 3F). In RCC tumors, gs_Meylan, gs_Chemokine, and infiltration were associated with better overall survival but worse ICB response, which indicated that patient survival outcomes are not solely determined by the degree of immune system activation [49] (Figure 3H). Of note, TLS-related signatures show a strong correlation with each other in both pan-cancer and cancer subgroups; however, they were negatively correlated with COX_IS and IPRES (Supplementary Figure S2). These results indicate a strong heterogeneity in the prognostic ability of the tumor markers we studied across different tumor types. Even features that exhibit significant prognostic capability at the pan-cancer level may show opposite trends in different tumor types. This variability is likely closely related to the malignancy degree and immune infiltration of different tumors.

### 3.3. Meta Analysis Identified a De Novo Pan-Cancer TLS Gene Signature Associated with ICB Response

**Figure 4:**
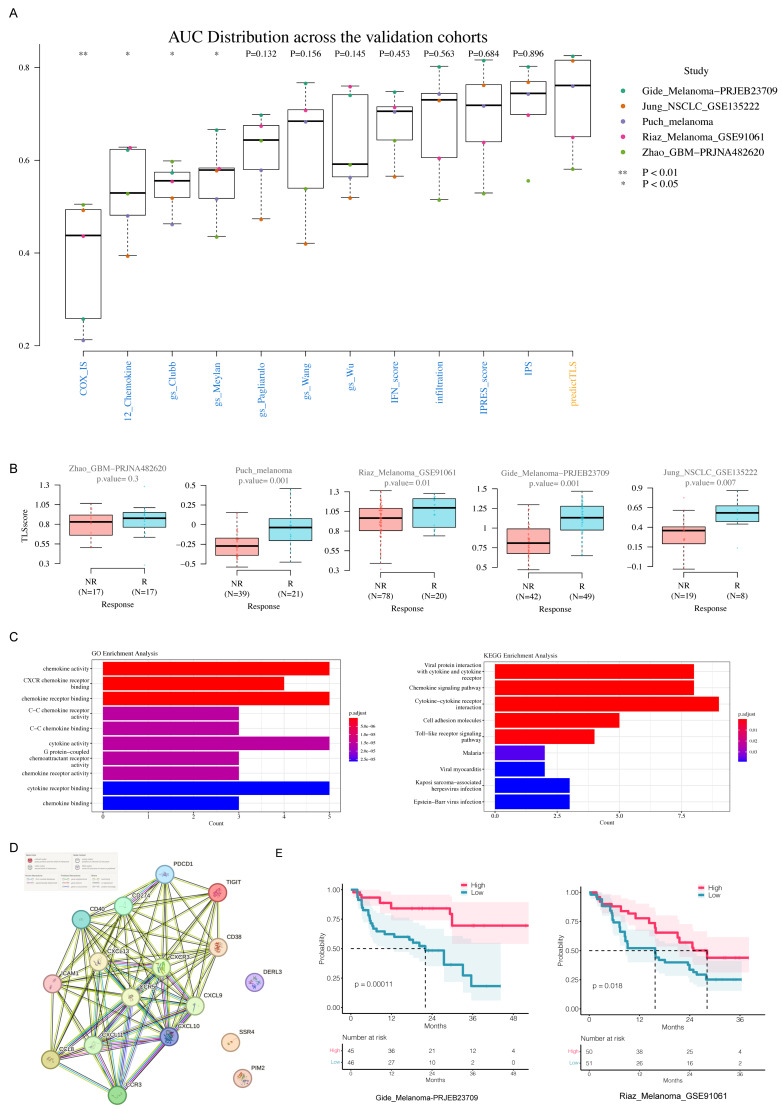
De novo TLS gene construction and validation in 5 validation ICB datasets. (**A**) AUC distribution across the validation datasets of 12 gene features and predictTLS. (**B**) PredictTLS is significantly associated with ICB response in 5 validation ICB datasets. (**C**) Gene ontology and KEGG enrichment analysis of 17 genes from predictTLS. (**D**) Protein and protein network analysis of 17 genes from predictTLS. (**E**) Kaplan–Meier plots of overall survival difference between tumors with high predictTLS score and low groups in dataset Gide_Melanoma-PRJEB23709.

Next, we seek to identify a de novo pan-cancer TLS gene signature, which could be more significantly associated with ICB response than existing ones. First, as the sizes of the ICB datasets we collected were uneven, we performed artificial partitioning of ICB cohorts to ensure that both the training and testing sets included both large and small datasets simultaneously. As a result, the discovery cohort comprised 10 datasets, while the validation cohorts consisted of 5 datasets (Supplementary Table S2A). Then we carried out a de novo meta-analysis of the 74 genes from six TLS gene signatures, which were present in every discovery cohort. We ranked the genes based on their logOR and derived signatures from the cutoff 0 up to cutoff 0.5 with a step size of 0.005 to investigate their association with IR. Finally, we selected 0.145 as the best cut-off; this list, containing 17 genes, is hereafter referred to as the PredictTLS signature (Supplementary Table S4). We then evaluated the predictive value of the PredictTLS signature. We defined the PredictTLS signature as the GSVA signatures composed of 17 genes (Figure 1, middle panel). The robustness of the PredictTLS gene signature was further validated in five independent cohorts of ICB-treated cancer patients, encompassing 313 patients with melanoma, GBM, and NSCLC. Remarkably, when compared to other candidate GE signatures, PredictTLS was the most robust predictor of IR, with significantly higher AUC values than three gene signatures (12_chemokine, gs_Clubb, gs_Meylan) and marginally significantly higher than the other three gene signatures (Figure 4A,B). Enrichment analysis for gene ontology and KEGG showed that these genes were associated with chemokine activity, CXCR chemokine receptor binding, and cytokine–cytokine receptor interaction (Figure 4C,D). In cohorts with available survival data, PredictTLS further exhibited a strong and significant association with OS (Figure 4E). Taken together, our results highlight the robust prognostic value of the PredictTLS gene signature and its potential clinical relevance as compared to the current standard.

### 3.4. Pan-Cancer TLS Gene Signature Is Associated with ICB Response (IR) in Single-Cell Transcriptome Data Across Various Cancer Types

**Figure 5:**
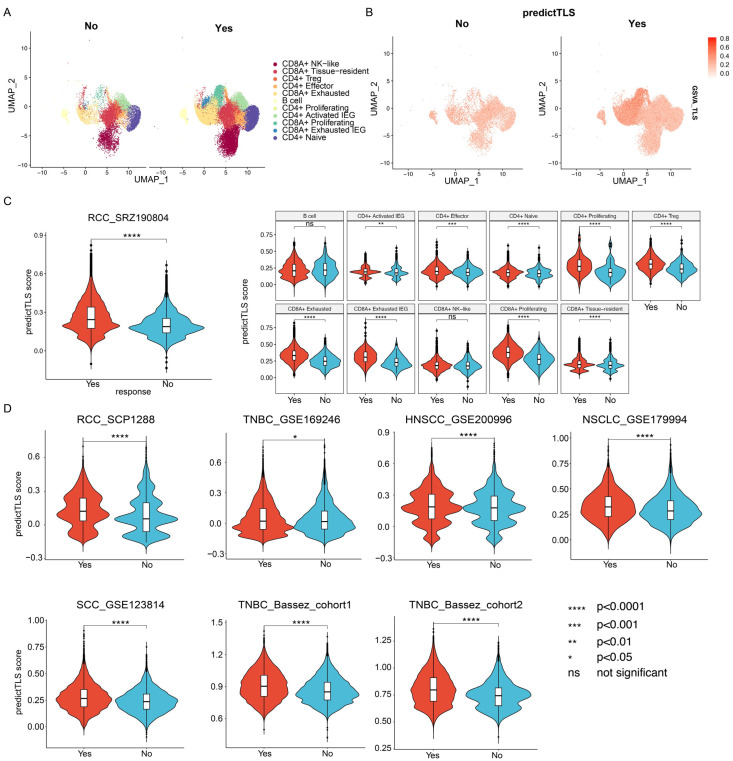
PredictTLS is significantly associated with ICB response in 8 scRNAseq datasets. (**A**) Umap of cell type distribution of ICB responder and non-responder in the RCC_ SRZ190804 dataset. (**B**) Featureplot of predictTLS signature score distribution of ICB responder and non-responder in RCC_SRZ190804 dataset. (**C**) PredictTLS is significantly associated with ICB response in RCC_SRZ190804 dataset, both on total cells or cell subpopulations. (**D**) PredictTLS is significantly associated with ICB response in the other 7 scRNAseq datasets across several cancer types.

As single-cell sequencing data has higher resolution, we further explored whether PredictTLS was associated with IR in single-cell transcriptome data from various cancer types (Figure 1, bottom panel). Firstly, we tested a single-cell dataset of renal cell carcinoma (RCC) from patients who received ICB immunotherapy, as RCC has been demonstrated to be rich in TLSs. Our results indicate that the immunotherapy-responsive group has more immune cell infiltration, including cytotoxic CD8 T cells, B cells, and other crucial components of TLSs, compared to the non-responsive group (Figure 5A,B). Additionally, PredictTLS is significantly correlated with immunotherapy efficacy. When analyzed according to cell subtypes, similar conclusions were observed (no significant differences in B cells and NK-like CD8 T cells) (Figure 5C). Subsequently, we further validated our findings in more tumor types and additional single-cell immunotherapy datasets. The results show a significant correlation between PredictTLS and immunotherapy efficacy in various solid tumors such as TNBC, HNSCC, NSCLC, and SCC (Figure 5D). Thus, these results further validate the reliability of predictTLS in predicting the efficacy of immunotherapy at the single-cell level.

### 3.5. PredictTLS Predicts Mature Tertiary Lymphoid Structures in TLS-Rich Carcinoma in Spatial Transcriptome Data

**Figure 6:**
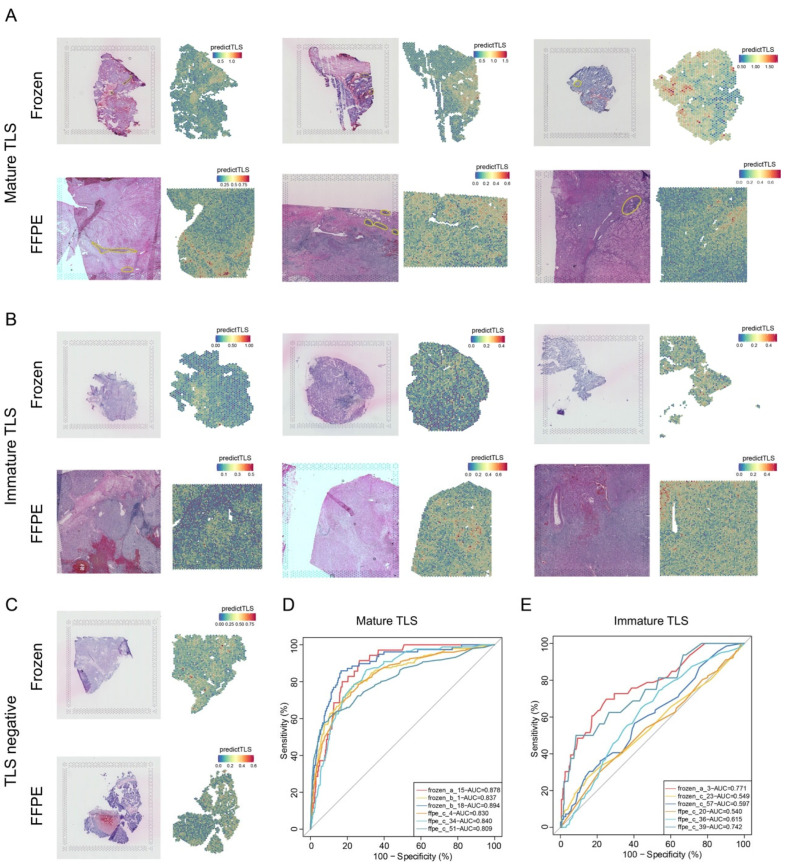
PredictTLS predicts mature tertiary lymphoid structures in TLS-rich carcinoma. (**A**) H&E images and spatial plot of predictTLS signature score in 3 frozen tissue sections and 3 FFPE tissue sections with mature TLSs. (**B**) H&E images and spatial plot of predictTLS signature score in 3 frozen tissue sections and 3 FFPE tissue sections with immature TLSs. (**C**) H&E images and spatial plot of predictTLS signature score in 1 frozen tissue section and 1 FFPE tissue section without TLS. (**D**) AUC distribution of predictTLS in TLS prediction across 6 samples with mature TLSs. (**E**) AUC distribution of predictTLS in TLS prediction across 6 samples with immature TLSs. The area circled in yellow represents the mature TLS region.

Spatial transcriptomics enables the localization and differentiation of the active expression of functional genes within specific tissue regions. We finally checked whether PredictTLS could predict mature TLSs based on spatial transcriptomics data in TLS-rich carcinoma (Figure 1, bottom panel). In total, 24 ccRCC primary tumors, using frozen (*n* = 12) and FFPE sections (*n* = 12) with spatial transcriptomics data, were grouped into three groups, mature TLSs, immature TLSs, and TLS-negative, based on TLS annotations retrieved from the original study. In six samples labeled as mature TLS, distinct tertiary lymphoid structures were visibly observed in the tissue slices. The PredictTLS scores for each spot in spatial transcriptomics showed a strong correspondence with the presence of tertiary lymphoid structures (Figure 6A). PredictTLS achieved an area under the curve (AUC) ranging from 0.809 to 0.894 in samples with mature TLSs (Figure 6D). However, in samples labeled as immature TLSs, typical tertiary lymphoid structures were not observed in the tissue slices, and there was no evident enrichment of PredictTLS scores in spatial transcriptomics spots (Figure 6B). PredictTLS could only reach an AUC ranging from 0.540 to 0.771 in samples with immature TLSs (Figure 6E). Similarly, in TLS-negative samples, there was no noticeable enrichment of PredictTLS scores in spatial transcriptomics spots, and the scores were generally low (Figure 6C). These results suggest that PredictTLS can accurately predict mature tertiary lymphoid structures in TLS-rich carcinoma.

## 4. Discussion

Tertiary lymphoid structures (TLSs) are located and develop in tissues that undergo antigen persistence, as seen in autoimmune diseases, chronic inflammation, chronic infections, graft rejection, and various cancers. The presence and density of intra-tumoral TLSs have been extensively demonstrated to be correlated with a favorable prognosis in many cancer types. Due to limited access to pharmacogenomic data for immunotherapies, assessing the validity and reproducibility of predictive TLS gene expression features for clinical outcomes remains an ongoing challenge. In this study, we conducted a comparative analysis to date of transcriptomic TLS-related biomarkers in the TCGA cohort and a pan-cancer cohort comprising over 1500 patients treated with immune checkpoint blockade. We first collected six representative immune-related features, such as TMB, and six TLS-related gene sets from publicly published literature. We conducted a benchmark analysis of these gene signatures on the TCGA dataset, and the benchmark results indicated that TMB could not effectively predict patients’ clinical prognosis at the pan-cancer level. Specifically, high TMB showed significant prognostic ability in melanoma and non-small cell lung cancer, which could be explained as tumors with a higher TMB and a higher number of mutations, which means the body’s immune system is more likely to identify cancer cells. While low TMB showed a close association with better prognosis in tumor types such as LIHC, KICH, THYM, GBM, and THCA, meta-analysis results also validated these conclusions, which were consistent with previous studies [50]. For instance, GBM patients with very low TMB have longer survival after immunotherapy. This paradoxical dependency may be a reflection of the immune-privileged environment of the central nervous system (CNS) [51].

Impressively, well-established immune markers, including COX_IS and IPRES, effectively predicted patients’ survival at the pan-cancer level. COX_IS is a signature capturing both ‘cancer-promoting’ and ‘cancer-inhibitory’ inflammatory. Bonavita et al. have revealed that the COX-IS is an independent prognostic factor across various cancer types, including HNSC, TNBC, MSKCM, KIRC, and OV, and uses Cox proportional hazards regression models. In this study, they also demonstrated that COX_IS predicts response to ICB in different tumor types, including melanoma, bladder cancer, renal cancer, and gastric cancer. However, in our study, we did not observe this significance, which could have resulted from the unevenness of tumor sample size; therefore, more datasets are needed to verify whether COX_IS can predict ICB efficacy at the pan-cancer level. In addition, for the first time, our study revealed that IPRES, a transcriptional signature related to innate anti-PD-1 resistance, could not predict survival or ICB efficacy at the pan-cancer level.

Similar to TMB, TLS-related gene features also lacked the ability to predict clinical prognosis at the pan-cancer level. In this study, the gene feature predictTLS obtained from the meta-analysis can effectively predict ICB response at the pan-cancer level. PredictTLS consists of 17 genes, including CCL8, CXCL13, and other chemokines, which play important roles in the formation of TLSs, fitting within the existing biological framework. CD38 and CD40 are important markers on the surface of B cells, consistent with the enrichment of B cells in TLSs. ICAM-1 belongs to the immunoglobulin superfamily and is a cell surface glycoprotein and adhesion receptor known for regulating the recruitment of white blood cells from circulation to sites of inflammation, participating in cell signaling and activation, immune responses, inflammation, and other important physiological processes. Additionally, predictTLS also includes two tumor immune checkpoint molecules, PDCD1 and TIGIT, which are consistent with previous reports of better response to ICB therapy in patients with high PD1/PD-L1 expression. We validated the robustness of predictTLS on five test datasets, single-cell ICB datasets, and spatial transcriptomic data, which also effectively validated the rationale and effectiveness of this gene set.

To provide a biological perspective related to maturation, we performed a functional association analysis of the PredictTLS genes based on key molecular features linked to the dynamic process of TLS formation in existing literature. We established an association framework based on existing literature: (1) Genes associated with early recruitment/initiation: CCL8, CXCL9, CXCL10, CXCL11, CXCR3, CCR3, CCR5, and ICAM1. These molecules primarily mediate the chemotaxis and adhesion of Th1 cells, cytotoxic T cells, dendritic cells, eosinophils, etc., serving as the “recruitment signals” for initial TLS formation. (2) Genes associated with maturation/structure and functional maintenance: B cell follicle/Germinal center-related: CXCL13, CD40, CD38, DERL3 (associated with ER-associated degradation, highly expressed in plasma cells), and SSR4 (translocon component, linked to antibody secretion). (3) T cell zone/Immune regulatory module: PDCD1, TIGIT, and CD274, reflecting the active network of T cell immune checkpoint regulation within the TLS. (4) Supportive cell/Stromal signals: PIM2 (a pro-survival kinase expressed in lymphocytes) may be involved in cell survival and proliferation.

In summary, we first systematically evaluated the association of six TLS gene signatures with immune-related indicators, as well as their predictive and prognostic performance in immune therapy. Subsequently, using meta-analysis methods, we constructed de novo TLS-related gene features that can effectively predict the efficacy of ICB therapy and prognosis. We validated the rationality and effectiveness of this de novo gene signature using independent validation cohorts of patients treated with immune checkpoint blockade, single-cell transcriptome data, and spatial transcriptome data.

It should be noted that our study focused on the spatial transcriptomic validation of clear cell renal cell carcinoma (ccRCC) samples, which represents a study limitation in terms of immediate pan-cancer applicability. The rationale for choosing ccRCC as a proxy includes: (1) High TLS prevalence and maturity: ccRCC is one of the solid tumors most frequently associated with the spontaneous development of structured, tertiary lymphoid structures (TLSs) that often exhibit germinal centers, making it an ideal model to study mature TLS biology [21]. (2) Established immunotherapy context: As a highly immunogenic tumor with standard-of-care ICB therapy, ccRCC provides a clinically relevant context to validate a TLS-based biomarker. (3) Feasibility of ground truth: The availability of well-annotated spatial datasets with clear histological TLS annotations for ccRCC was crucial for performing a rigorous, gold-standard validation of our PredictTLS score against pathologist-defined TLS regions.

While histopathological assessment of TLSs, based on H&E or multiplex immunofluorescence staining of consecutive tissue sections, remains the gold standard for spatial and cellular characterization, our computational PredictTLS score offers several complementary advantages for translational and clinical research. First, it provides an objective, continuous, and quantitative metric derived from bulk or spatial transcriptomics, reducing observer variability inherent in semi-quantitative pathological grading systems. Second, PredictTLS can be applied at scale to archival samples and existing transcriptomic datasets where full tissue sections or multiplex staining are unavailable, enabling retrospective analyses across large, multi-center cohorts. Most importantly, it may serve as an efficient digital pre-screening tool to identify cases with high TLS likelihood for subsequent focused, in-depth histological or spatial multi-omics validation, thereby optimizing resource use. Thus, PredictTLS does not replace but rather extends traditional pathology, offering a reproducible, high-throughput companion for biomarker development and patient stratification in immunotherapy studies.

Our study has several limitations. First, the number of collected ICB immunotherapy datasets and the coverage of tumor types are still limited. Additionally, due to variations in sample sizes among datasets, there might be systematic bias during the training and testing set partitioning. Second, the number of TLS-related feature genes collected in this study is limited, with only 74 genes. Including more feature genes as TLS-related research progresses would help further optimize the predictTLS gene set obtained from prediction results. Third, it is necessary to validate and expand our conclusions in larger ICB datasets, single-cell datasets, and spatial transcriptomic datasets. Fourth, our study only focused on TLS-related transcriptomic features for biomarker development; integrative biomarkers combining transcriptomic, genomic, and microenvironmental features might enhance the generalizability and applicability of the biomarker [52,53,54]. Completing the aforementioned improvements will further help to identify predictive biomarkers of ICB efficacy based on tertiary lymphoid structures (TLSs), which is of crucial significance for understanding the role of TLSs in precision immunotherapy for cancer.

## Data Availability

All the code for reanalysis of results and figures is also available at https://github.com/ChiZhou0514/Pancancer_TLS.git (accessed on 26 January 2026). The TCGA and ICB cohorts’ transcriptome and clinical data are available at Zenodo with DOI: 10.5281/zenodo.18552287; the scRNAseq and spatial transcriptome were retrieved from original studies.

## Supplementary Materials

The following supporting information can be downloaded at: https://www.mdpi.com/article/10.3390/genes17020239/s1. Figure S1. Multivariate model and meta-analysis of all features in TCGA and ICB cohorts, related to Figure 2. (A) Multivariate model and meta-analysis of all features in TCGA “hot” and “cold” tumor cohorts. (B) Multivariate model and meta-analysis of all features in ICB cohorts. Figure S2. Spearman Correlation between 12 Gene signatures, illustrated by pan-cancer and cancer type. Table S1. Six TLS-associated signatures curated from Pubmed. Table S2. ICB and scRNAseq dataset information used in this study. Table S3. Multivariate Cox model and meta-analysis of all features in TCGA “hot” and “cold” tumor cohorts. Table S4. 17 PredictTLS genes derived from this study.

## Abbreviations

The following abbreviations are used in this manuscript:

TLSs	Tertiary lymphoid structures
TCGA	The Cancer Genome Atlas
ICB	Immune checkpoint blockade
SLO	Secondary lymphoid organs
PFS	Progression-free survival
OS	Overall survival
TMB	Tumor mutation burden
ccRCC	Clear cell renal cell carcinoma
GSVA	Gene Set Variation Analysis
LIHC	Liver Hepatocellular Carcinoma
KICH	Kidney Chromophobe
THYM	Thymoma
THCA	Thyroid Carcinoma
NSCLC	Non-Small Cell Lung Cancer
SKCM	Skin Cutaneous Melanoma
HRs	Hazard Ratios
