# Supplementary material for: Identification of a Tertiary Lymphoid Structure Signature for Predicting Tumor Outcomes Through Transcriptomics Analysis
Source: Genes (Basel). 2026 Feb 16;17(2):239. doi: 10.3390/genes17020239 (PMC12940614; doi:10.3390/genes17020239)
Supplement: Supplementary file 1 [file genes-17-00239-s001.zip › Supplementary Figure S1.pdf]

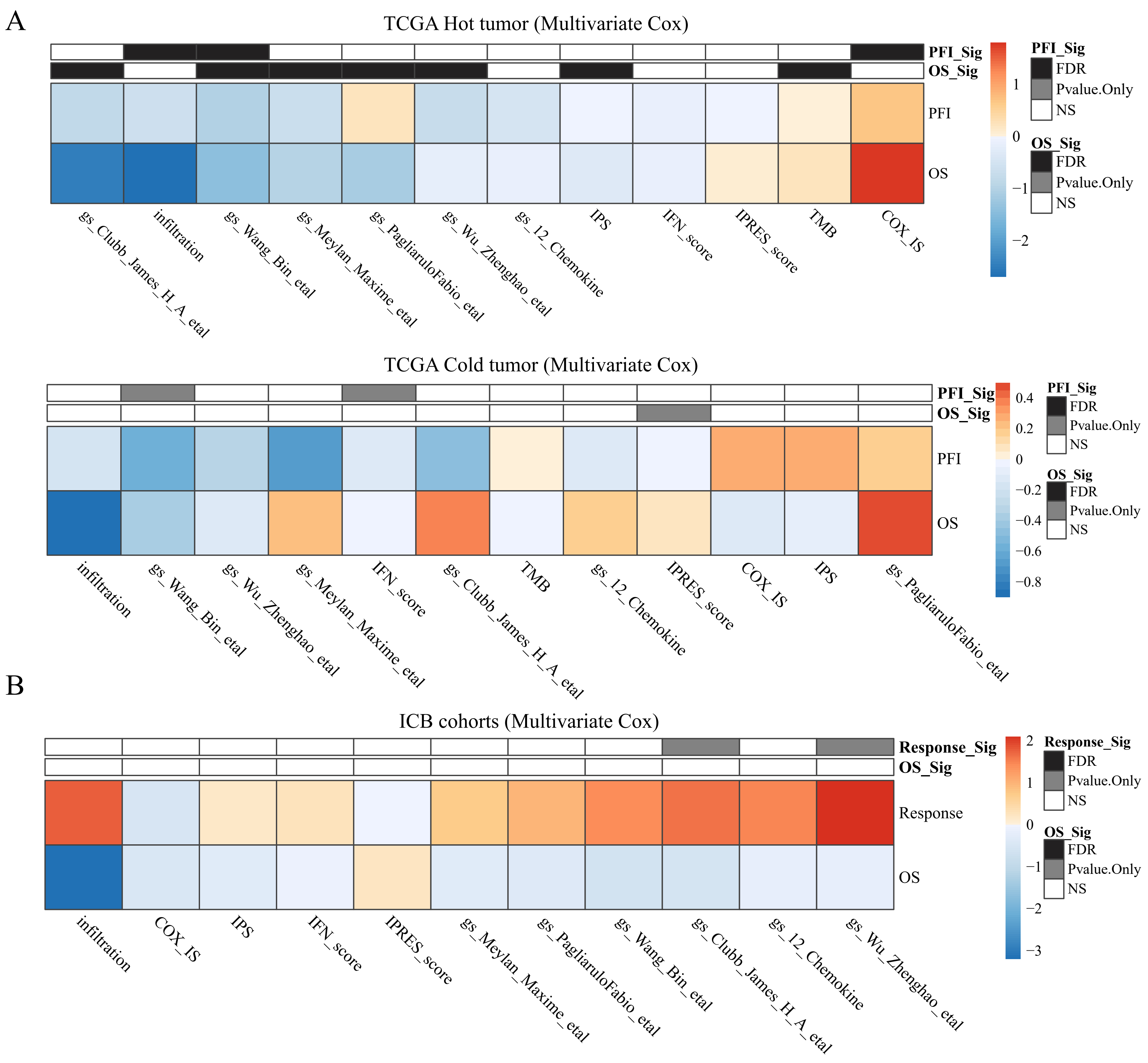
A
TCGA Hot tumor (Multivariate Cox)
PFI_Sig
OS_Sig
PFI
OS
gs_Clubb_James_H_A_etal
infiltration
gs_Wang_Bin_etal
gs_Meylan_Maxime_etal
gs_PagliaruloFabio_etal
gs_Wu_Zhenghao_etal
gs_12_Chemokine
IPS
IFN_score
IPRES_score
TMB
COX_IS
1
0
−1
−2
PFI_Sig
FDR
Pvalue.Only
NS
OS_Sig
FDR
Pvalue.Only
NS
TCGA Cold tumor (Multivariate Cox)
PFI_Sig
OS_Sig
PFI
OS
infiltration
gs_Wang_Bin_etal
gs_Wu_Zhenghao_etal
gs_Meylan_Maxime_etal
IFN_score
gs_Clubb_James_H_A_etal
TMB
gs_12_Chemokine
IPRES_score
COX_IS
IPS
gs_PagliaruloFabio_etal
0.4
0.2
0
−0.2
−0.4
−0.6
−0.8
PFI_Sig
FDR
Pvalue.Only
NS
OS_Sig
FDR
Pvalue.Only
NS
B
ICB cohorts (Multivariate Cox)
Response_Sig
OS_Sig
Response
OS
infiltration
COX_IS
IPS
IFN_score
IPRES_score
gs_Meylan_Maxime_etal
gs_PagliaruloFabio_etal
gs_Wang_Bin_etal
gs_Clubb_James_H_A_etal
gs_12_Chemokine
gs_Wu_Zhenghao_etal
2
1
0
−1
−2
−3
Response_Sig
FDR
Pvalue.Only
NS
OS_Sig
FDR
Pvalue.Only
NS
