# Supplementary material for: Identification of a Tertiary Lymphoid Structure Signature for Predicting Tumor Outcomes Through Transcriptomics Analysis
Source: Genes (Basel). 2026 Feb 16;17(2):239. doi: 10.3390/genes17020239 (PMC12940614; doi:10.3390/genes17020239)
Supplement: Supplementary file 1 [file genes-17-00239-s001.zip › Supplementary Figure S2.pdf]

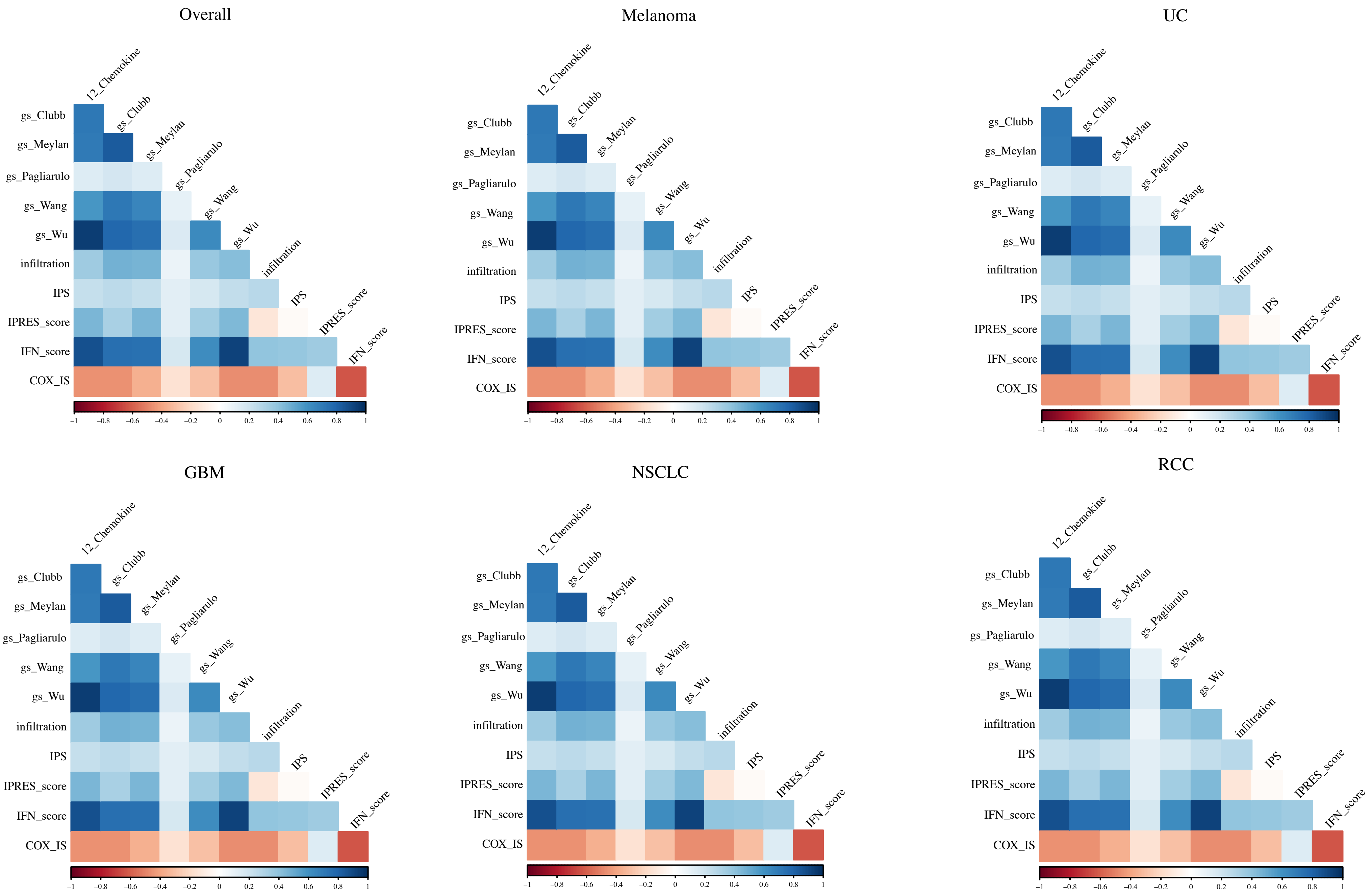
Overall
Melanoma
UC
GBM
NSCLC
RCC
12_Chemokine
gs_Clubb
gs_Meylan
gs_Pagliarulo
gs_Wang
gs_Wu
infiltration
IPS
IPRES_score
IFN_score
COX_IS
−1
−0.8
−0.6
−0.4
−0.2
0
0.2
0.4
0.6
0.8
1
